# Orbital and Maxillofacial Soft Tissue Infection Caused by Methicillin-Resistant *Staphylococcus aureus* With Diabetic Ketoacidosis in a Young Man: A Case Report

**DOI:** 10.1155/carm/9977753

**Published:** 2025-09-01

**Authors:** Jinhui Yao, Yujing Zhan, Chuanying Zhu, Xiaojuan Wang, Hongmei Kang, Ting Zhao

**Affiliations:** ^1^Oral and Maxillofacial Surgery, Shandong Provincial Hospital Affiliated to Shandong First Medical University, Jinan, China; ^2^Department of Medical Imaging, Cancer Hospital of Shandong First Medical University, Jinan, China; ^3^Outpatient Department, Shandong Provincial Hospital of Traditional Chinese Medicine, Jinan, China

## Abstract

Oral and maxillofacial space infection (OMSI) progresses rapidly, and when combined with diabetic ketoacidosis (DKA), it can become a serious and life-threatening condition. Cases of OMSI with concurrent DKA are relatively rare. This case report describes a young man who developed OMSI caused by methicillin-resistant *Staphylococcus aureus* in the setting of DKA. A 21-year-old man presented with swelling and pain in the right lower lip that had persisted for four days; the symptoms were both atypical and severe. Based on the patient's clinical course, hyperglycemia may play an important role in the onset and progression of OMSI in young individuals. It is essential to identify the underlying cause of OMSI and to closely monitor clinical signs for timely intervention.

## 1. Introduction

Oral and maxillofacial space infection (OMSI) is an infectious condition that affects the deep fascial spaces of the maxillofacial region. Due to the rich vascular supply and anatomical interconnectivity between soft tissue compartments, inflammation can progress rapidly. Superior spread may result in intraorbital or intracranial infections, while inferior spread can lead to airway obstruction, mediastinitis, or pneumonia. In severe cases, systemic dissemination via the bloodstream may cause sepsis or toxic shock [1, 2]. The mortality rate of OMSI is estimated to range between 10% and 40% [3]. Diabetic ketoacidosis (DKA) is a serious and potentially life-threatening acute complication of diabetes. In patients with concurrent OMSI and DKA, metabolic instability may exacerbate infection symptoms, making the condition more severe and difficult to manage, with an increased risk of fatality [4]. Currently, reports on OMSI cases complicated by DKA are limited, and clinical experience, particularly in nursing care, remains insufficient.

In this retrospective case report, we describe our experience managing a young man with OMSI caused by multidrug-resistant bacteria, complicated by severe DKA, who was admitted to Shandong Provincial Hospital (Jinan, China) in November 2023. The infection developed rapidly due to a lip skin injury and presented with atypical clinical manifestations. Upon admission, an accurate diagnosis was made, followed by timely medical and nursing interventions. The patient responded well to treatment. This case may serve as a reference for healthcare professionals managing similar presentations.

## 2. Case Presentation

**Figure 1:**
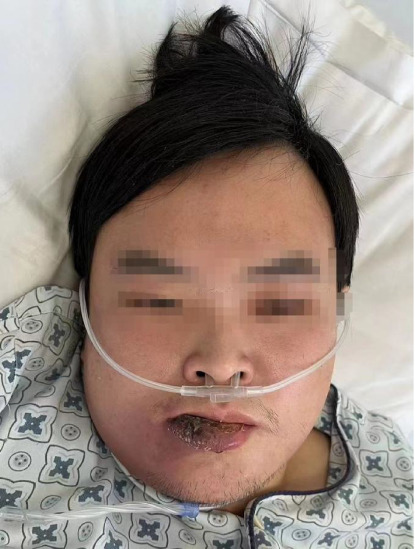
The patient upon admission shows a skin injury in the right lower lip and swelling of the right jaw.

A 21-year-old man was admitted to the hospital with swelling and pain in the right lower lip that had persisted for four days (Figure 1). Four days before admission, the patient experienced right lip pain and swelling following a skin injury. He self-administered anti-inflammatory medication, which was ineffective. He was unaware of his elevated blood glucose levels and had not been monitoring them.

Three days later, he developed chest tightness and shortness of breath. Maxillofacial computed tomography (CT) revealed marked swelling and thickening of the soft tissue in the right lip and submandibular region, with increased density and irregular skin surface. Grid-like changes were observed subcutaneously in the right maxillofacial area. Additionally, multiple lymph nodes of varying sizes were detected around the carotid sheath and beneath the maxilla. The largest lymph nodes were located in the right mandibular region, with a maximum short-axis diameter of approximately 1.1 cm. Subsequently, the patient was admitted to the Department of Oral and Maxillofacial Surgery for treatment. After hospital admission, his level of consciousness gradually declined from wakefulness to confusion, accompanied by shortness of breath. Physical examination findings on admission were as follows: temperature, 37.1°C; heart rate, 121 beats per minute; respiratory rate, 29 breaths per minute; oxygen saturation (SpO_2_), 96%; blood pressure, 132/78 mmHg; and body mass index, 24.72. The patient presented with facial asymmetry, swelling of the right lower lip and right jaw, and moderate firmness on palpation without obvious fluid fluctuation. Maximum mouth opening was approximately two fingers. Laboratory tests conducted after admission revealed severe metabolic acidosis with respiratory alkalosis. The highest recorded blood glucose level was 30.21 mmol/L. The patient was diagnosed with OMSI complicated by DKA. Blood culture revealed Gram-positive cocci, while culture of oral secretions identified *Staphylococcus aureus*, which was confirmed to be multidrug-resistant.

Treatment was initiated immediately based on clinical and laboratory findings. The patient received symptomatic and supportive care, including oxygen therapy via mask, intravenous fluid resuscitation, gastric acid suppression, anti-inflammatory therapy, expectorants, correction of acid–base imbalance, and blood glucose control. Based on the results of the bacterial culture, the initial empirical antibiotics (piperacillin–sulbactam) were adjusted to a regimen including vancomycin, a high-grade antibiotic, combined with ceftazidime.

After 21 days of hospitalization, the swelling in the right jaw had significantly decreased, and the patient appeared to be in good spirits. Maxillofacial CT re-examination revealed that the extent of soft tissue infection in the right maxillofacial region had reduced compared with previous imaging. Blood cultures were negative on two consecutive occasions, and blood glucose levels stabilized between 6.1 and 7.9 mmol/L. The patient was subsequently discharged. During an outpatient follow-up visit 3 months later, the patient's condition remained stable. Blood glucose levels were well controlled, and he had resumed normal academic and daily activities.

## 3. Discussion

### 3.1. Correction of Acid–Base Imbalance

**Figure 2:**
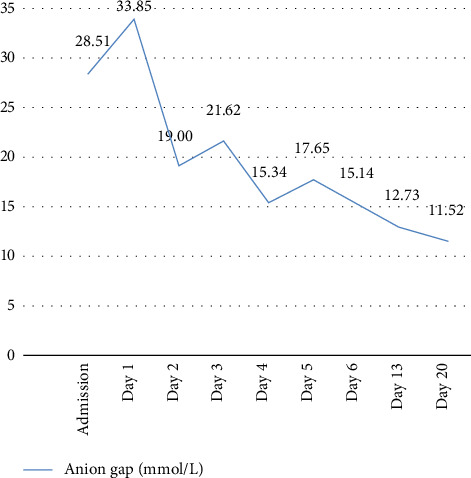
Anion gap analysis results at key points during hospitalization. Reference range: 12–20 mmol/L.

Upon hospital admission, the patient's blood test revealed an anion gap (AG) of 28.51 mmol/L, suggesting the presence of an acid–base imbalance. The AG is an important marker for assessing disturbances in the acid–base status of body fluids. It aids in diagnosing potentially life-threatening conditions and provides critical information for evaluating patient status [5]. An urgent arterial blood gas analysis was performed with the following results: pH, 7.08; PCO_2_, 12 mmHg; blood glucose, 24.8 mmol/L; and serum sodium, 119.00 mmol/L. In addition, urinalysis showed strongly positive ketone bodies (80 mg/dL). Based on these findings, the patient was diagnosed with DKA with acid–base disturbance—specifically, severe metabolic acidosis accompanied by respiratory alkalosis. The patient was immediately administered intravenous 5% sodium bicarbonate to correct the acidosis and stabilize the internal environment. Due to poor oral intake, a nasogastric tube was placed for enteral feeding to ensure adequate hydration. According to the endocrinology consultation, daily fluid intake was required to exceed 3000 mL. Continuous monitoring of the patient's arterial blood gases was essential for maintaining water, electrolyte, and acid–base balance. On the second day of admission, the urine ketone test remained positive, although the AG value had returned to normal. The normalization of the AG value preceded the clearance of urinary ketones, which became negative on the sixth day. This finding indicates that the AG value may serve as an early marker of treatment efficacy in patients with DKA (Figure 2).

Arterial blood gas analysis on admission revealed a serum sodium level of 119.00 mmol/L. Sodium supplementation was administered both intravenously and orally. Simultaneously, serum sodium levels were closely monitored to avoid overly rapid correction, which may lead to osmotic demyelination syndrome (Table 1). Clinicians should also be attentive to early signs of potassium metabolism disorders. In most cases, the risk of aggravating acidosis is lower than the risk of worsening hypokalemia, which can result in life-threatening arrhythmias [6]. As the patient was in a state of high potassium consumption and unable to ingest food orally, serum potassium levels required close monitoring. Potassium supplementation therapy was administered accordingly (Table 1).

### 3.2. Strict Blood Glucose Management

The patient was unaware of having diabetes. Upon admission, his blood glucose level was 30.21 mmol/L, and glycated albumin was 33.1% (reference range: 4%–6%), indicating long-term hyperglycemia. This finding suggests that chronic hyperglycemia may play a significant role in the progression of inflammation.

Following the endocrinology consultation, blood glucose was closely monitored and insulin therapy adjusted accordingly. The patient received intravenous normal saline and insulin via micropump. When blood glucose dropped below 13.9 mmol/L, a glucose–insulin regimen was administered through the micropump. In addition, 12 units of insulin glargine were administered each night to maintain target fasting blood glucose levels below 11.1 mmol/L [7]. Insulin dosage was adjusted according to the patient's blood glucose levels. The frequency of blood glucose monitoring was modified based on clinical status—initially every 2 hours, then reduced to seven times per day (before each meal, 2 hours after meals, and at bedtime). Once blood glucose levels stabilized, monitoring was performed twice daily (fasting and bedtime). Simultaneously, clinicians closely monitored urinary ketone levels via routine urinalysis. On the sixth day after hospital admission, urinary ketones became negative.

### 3.3. Effective Treatment of Maxillofacial Space Infection

Maxillofacial space infections can progress rapidly. If not promptly controlled and effectively treated, they may result in life-threatening complications [8]. The patient's vital signs were closely monitored, with particular attention to respiratory rate, SpO_2_, and level of consciousness. Symptomatic treatment was administered promptly when abnormal signs or severe dyspnea were observed. Electrocardiographic monitoring and continuous low-flow oxygen therapy were also provided. When the patient's level of consciousness changed from wakefulness to confusion, clinicians evaluated the underlying cause based on clinical examination findings.

The patient exhibited swelling of the oral and maxillofacial soft tissues, disruption of the skin–mucosal barrier, and a high likelihood of secondary infection due to oral flora. Furthermore, the presence of hyperglycemia and reduced immune function made the infection more difficult to control. To prevent further deterioration, the patient was advised to maintain oral hygiene and received oral irrigation twice daily.

After admission, relevant etiological examinations were conducted to identify the types of infecting microorganisms. Due to noticeable swelling of the right lower lip with purulent discharge, a sample of local secretions was sent for bacterial culture. Sputum and blood cultures were also performed to determine whether pulmonary or hematogenous infections were present. According to the consultation with the infectious disease department, antibiotic therapy was initiated empirically, strictly following the physician's recommendations. The patient was treated with piperacillin–sulbactam and metronidazole. Culture of the local secretions identified *S.* aureus (MRSA), and Gram staining of the blood culture detected Gram-positive cocci. MRSA bloodstream infections account for 10%–40% of cases and are associated with high mortality [9]. Vancomycin, a high-grade antibiotic specifically indicated for MRSA, was promptly initiated. The dosage was calculated based on the patient's creatinine clearance rate, and clinicians closely monitored renal function [10]. Additionally, serum vancomycin concentrations were monitored, and the dosage was adjusted accordingly. Clinicians were also advised to remain vigilant for possible Gram-negative bacilli infection; therefore, anti–Gram-negative therapy with ceftazidime was coadministered.

### 3.4. Control of Multidrug-Resistant Infection Spread

**Figure 3:**
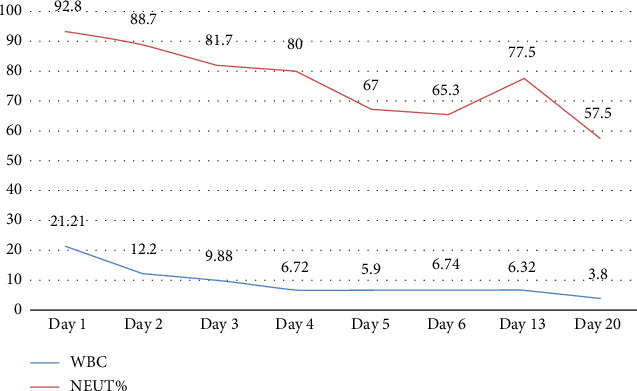
WBC and NEUT% results at key points during hospitalization. WBC: white blood cell count (reference range: 3.5–9.5 × 10^9^/L); NEUT%: neutrophil percentage (reference range: 40%–75%).

**Figure 4:**
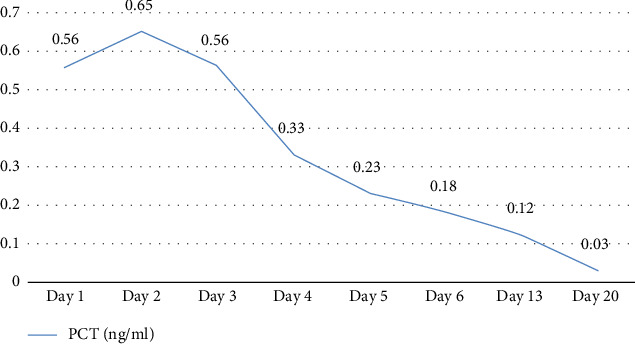
PCT results at key points during hospitalization. PCT: procalcitonin (reference range: 0–0.05 ng/mL).

**Figure 5:**
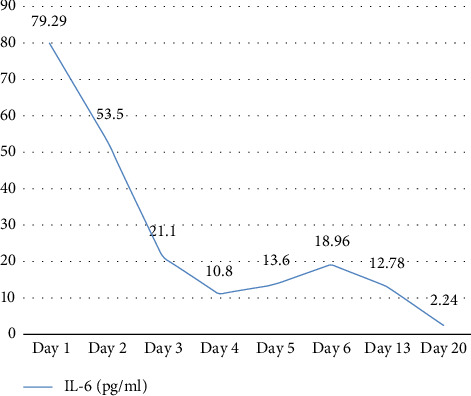
IL-6 results at key points during hospitalization. IL-6: interleukin-6 (reference range: 0–7 pg/mL).

As the patient was infected with MRSA, strict disinfection and isolation measures were implemented to prevent transmission. The following precautions were taken: The ward was ventilated twice daily by opening windows; bedside isolation was maintained, with isolation signage displayed prominently next to the patient's bed; medical staff adhered to strict hand hygiene and aseptic protocols and used appropriate personal protective equipment. Patient-specific equipment was used, and frequently touched surfaces were disinfected with a chlorine-containing solution at a concentration of 1000 mg/L. Medical waste was placed in double-layered, leak-proof yellow bags, sealed promptly, and labeled as infectious waste. The patient's body temperature and infection-related biomarkers were closely monitored. These included white blood cell count (WBC), neutrophil percentage (NEUT%), interleukin-6 (IL-6), and procalcitonin (PCT). These indicators not only assist in assessing disease severity but also reflect the effectiveness of anti-infective treatment [11, 12] (Figures 3, 4, 5).

### 3.5. Control of Pulmonary Infection

Chest CT of the patient revealed multiple nodules in both lungs, and hemorrhagic dissemination of *S. aureus* to the lungs was suspected. In accordance with medical recommendations, the patient was treated with nebulized inhalation therapy and expectorants. Clinicians also guided the patient in effective coughing and sputum expectoration. Serial chest CT scans were performed to monitor disease progression and evaluate the effectiveness of anti-infective treatment.

### 3.6. Individualized Nutritional Support

To enhance the efficacy of antibiotics in patients with severe OMSI, attention must be given to serum albumin levels, as hypoproteinemia can affect antibiotic binding capacity. The patient's albumin level at admission was 49.4 g/L, but it declined to 32.9 g/L at 10:00 p.m. on the second day. Albumin supplementation was promptly administered to correct hypoproteinemia.

Initially, the patient was unable to consume food orally and was therefore provided with nasogastric feeding to ensure adequate enteral nutrition. This was combined with parenteral nutritional support to prevent negative nitrogen balance. On the sixth day, as the patient's condition improved, the gastric tube was removed and oral feeding was resumed. Simultaneously, diabetic dietary counseling was provided to prevent hypoglycemia. Upon discharge, the patient's serum albumin level had returned to normal.

### 3.7. Psychological Nursing

The patient was a young man who experienced an acute disease onset. Clinicians explained the cause, treatment options, and prognosis to the patient in a timely manner to improve his understanding of the condition, alleviate anxiety and fear, and enhance treatment compliance. In addition, communication with family members was encouraged to provide emotional support and motivate the patient. As a result, the patient remained emotionally stable and actively cooperated with medical treatment.

## 4. Conclusion

For patients with OMSI, early identification of underlying triggers and prompt, effective treatment are essential to limit disease progression and prevent serious complications. In this case, the patient—a young man—was admitted due to a lip skin injury. Had he monitored his blood glucose levels regularly, it is likely that the acute infection and DKA could have been avoided. This case highlights that hyperglycemia may play a significant role in the onset and progression of OMSI among younger individuals. Therefore, in young and middle-aged patients presenting with acute space infections—especially with atypical symptoms and rapid progression—blood glucose monitoring and control should be emphasized. Early identification and intervention can delay disease progression and improve clinical outcomes in such patients.

This case involved a young man who developed severe OMSI caused by MRSA, accompanied by DKA—a rare but clinically significant presentation with a favorable prognosis. As such, it provides valuable reference material for clinical diagnosis and treatment. Severe OMSI represents an important public health concern [13]. Existing studies have demonstrated a close association between hyperglycemia and OMSI. However, this case revealed that public awareness of OMSI is limited—particularly regarding the role of blood glucose levels in disease progression. Therefore, it is essential to strengthen health education efforts, disseminate relevant information, and improve public understanding of OMSI and glycemic control, especially among younger individuals. These measures may help prevent the progression of OMSI to more severe stages.

## Figures and Tables

**Table 1:** Laboratory results of the patient during hospitalization.

Day	Blood glucose (reference: 3.9–6.1 mmol/L)	Serum albumin (reference: 40–55 g/L)	Serum sodium (reference: 137–147 mmol/L)	Serum potassium (reference: 3.5–5.5 mmol/L)
Day 1	29.36	49.4	122.5	5.75
Day 2	12.10	32.9	131.3	3.59
Day 3	10.64	32.1	132.0	3.82
Day 4	11.36	35.6	135.0	4.12
Day 5	10.63	39.9	133.5	4.55
Day 6	12.00	42.8	132.3	4.44
Day 7	11.05	49.5	135.6	4.32
Day 11	10.76	/	136.0	4.57
Day 12	9.03	/	134.8	4.83

## Data Availability

The article is a case report written regarding a young patient who developed OMSI complicated by DKA. The data can be accessed upon request.
